# Is There a Relation between EEG-Slow Waves and Memory Dysfunction in Epilepsy? A Critical Appraisal

**DOI:** 10.3389/fnhum.2015.00341

**Published:** 2015-06-11

**Authors:** Yvonne Höller, Eugen Trinka

**Affiliations:** ^1^Department of Neurology, Christian Doppler Medical Centre and Centre for Cognitive Neuroscience, Paracelsus Medical University, Salzburg, Austria

**Keywords:** epilepsy, memory, slow waves, consciousness, sleep, seizure

## Abstract

Is there a relationship between peri-ictal slow waves, loss of consciousness, memory, and slow-wave sleep, in patients with different forms of epilepsy? We hypothesize that mechanisms, which result in peri-ictal slow-wave activity as detected by the electroencephalogram, could negatively affect memory processes. Slow waves (≤4 Hz) can be found in seizures with impairment of consciousness and also occur in focal seizures without impairment of consciousness but with inhibited access to memory functions. Peri-ictal slow waves are regarded as dysfunctional and are probably caused by mechanisms, which are essential to disturb the consolidation of memory entries in these patients. This is in strong contrast to physiological slow-wave activity during deep sleep, which is thought to group memory-consolidating fast oscillatory activity. In patients with epilepsy, slow waves may not only correlate with the peri-ictal clouding of consciousness, but could be the epiphenomenon of mechanisms, which interfere with normal brain function in a wider range. These mechanisms may have transient impacts on memory, such as temporary inhibition of memory systems, altered patterns of hippocampal–neocortical interactions during slow-wave sleep, or disturbed cross-frequency coupling of slow and fast oscillations. In addition, repeated tonic–clonic seizures over the years in uncontrolled chronic epilepsy may cause a progressive cognitive decline. This hypothesis can only be assessed in long-term prospective studies. These studies could disentangle the reversible short-term impacts of seizures, and the impacts of chronic uncontrolled seizures. Chronic uncontrolled seizures lead to irreversible memory impairment. By contrast, short-term impacts do not necessarily lead to a progressive cognitive decline but result in significantly impaired peri-ictal memory performance.

## 1 Introduction

Memory deficits cause severe impairment of quality of life in patients with epilepsy (Thompson and Corcoran, 1992; Motamedi and Meador, 2003; Elger et al., 2004; van Rijckevorsel, 2006; Butler and Zeman, 2008). It is widely acknowledged that memory is impaired in temporal lobe epilepsy (TLE) (Helmstädter, 2002; Helmstädter and Elger, 2009), and there is growing evidence that this is also the case in other forms of epilepsy such as frontal lobe epilepsy (Centeno et al., 2010), idiopathic occipital lobe epilepsy (Gülgönen et al., 2000), benign epilepsy with centrotemporal spikes (BECTS) (Verrotti et al., 2014), juvenile myoclonic epilepsy (Pascalicchio et al., 2007), and encephalopathy with status epilepticus during slow-wave sleep (Tassinari et al., 2009).

Different types of epilepsy come along with varying memory deterioration of different origin (Elger et al., 2004). In addition, since memory is not a unitary entity, different forms of epilepsy affect different memory functions, albeit in most cases, problems arise with long-term memory. Epilepsy is a heterogeneous group of disorders and each syndrome most likely reflects specific pathological changes in the brain (Duncan, 1997) and eventually a specific genetic background (Buono, 2013; Thomas and Berkovic, 2014), potentially affecting memory in addition to the seizures themselves. Moreover, anti-epileptic drugs often have memory-relevant adverse effects (Motamedi and Meador, 2004; Jokeit et al., 2005; Hermann et al., 2010; Laxer et al., 2014). In addition, psychosocial consequences of the disorder (Elixhauser et al., 1999; Spatt et al., 2005) with lower level of education and social integration may also play a role. Finally, seizures themselves are likely to affect memory (Jokeit et al., 2005), but the influence may depend on several factors, such as age, localization, type of seizure, or frequency (Elger et al., 2004; Haut et al., 2004; Thompson and Duncan, 2005; Vingerhoets, 2006; Stefan and Pauli, 2008; Helmstädter and Elger, 2009).

A seizure may occur in its focal form, originating within networks limited to one hemisphere, or generalized, when they occur in and rapidly engage bilaterally distributed networks (Berg et al., 2010). In addition, focal seizures may occur without impairment of consciousness or awareness (previously called “simple partial”) as well as with impairment of consciousness or awareness (previously called “complex partial” or “dyscognitive”) (ILAE, 1981; Blume et al., 2001; Berg et al., 2010). Disordered consciousness during or after a seizure, i.e., during the so-called post-ictal state, is a highly interesting phenomenon, but still, it is not well understood (Shorvon and Trinka, 2010). In contrast to the clear loss of consciousness in generalized tonic–clonic seizures leading to ictal and postictal coma, focal seizures, e.g., in TLE, may lead to various degrees of impairment of consciousness (Blumenfeld, 2003). In the network inhibition hypothesis, Englot and Blumenfeld (2009) proposed that peri-ictal sleep-like slow rhythms could mediate the impairment of consciousness in TLE.

*Is it possible, that the slow wave activity (* ≤*4 Hz) as measured by the electroencephalogram (EEG) is the link between seizures affecting consciousness and memory?* We wondered if there is any evidence for this speculation, that peri-ictal slow waves in the EEG are related to long- and short-term memory effects in different forms of epilepsy. For the purpose of this article, we define (1) *long-term memory effects* as a general – and in most cases irreversible – damage to the memory system in patients with epilepsy, causing impairment of memory functions. And (2) *short time effects* as transient mechanisms with seizure-related deletion of memory entries in the sense of forgetting, inaccurate consolidation, or a temporary block of access to memory functions. The purpose of this article is to critically discuss the potential relationship between memory functions, peri-ictal or sleep-related slow waves, and the peri-ictal loss of consciousness. In addition, we emphasize the difference between functional sleep-related and pathologic peri-ictal slow-wave activity. Finally, we will present some speculative hypotheses and suggest future areas of research in this article.

## 2 Peri-Ictal Loss of Consciousness and Memory

“Consciousness exists, but it resists definition” (Honderich, 1995). Thus, we only can operationalize the assessment of consciousness by (1) testing the responsiveness to external stimuli, which depends on wakefulness and awareness of the individual, and (2) recollection of presented stimuli, reflecting learning and memory (Baars, 1988; Lux et al., 2002).

Impaired consciousness or awareness is accompanied by electroencephalographic slow-wave activity, e.g., in peri-ictal loss of consciousness in patients with epilepsy (Blumenfeld, 2004; Englot and Blumenfeld, 2009; Englot et al., 2010), deep sleep (Evans, 2003), and specific forms of disorders of consciousness such as the apallic syndrome and its reorganizational stages (Kretschmer, 1940; Gerstenbrand et al., 1963), also known as vegetative state (or unresponsive wakefulness) and the minimally conscious state (Davey et al., 2000; Coleman et al., 2005; Kotchoubey et al., 2005; Leon-Carrion et al., 2008, 2009; Babiloni et al., 2009; Cimenser et al., 2011; Lehembre et al., 2012; Fingelkurts et al., 2013; Höller et al., 2013, 2014). Indeed, patients with disorders of consciousness can be differentiated on a single-subject level from conscious individuals by connectivity patterns in slow EEG frequencies at rest (Höller et al., 2014).

It became clear that memory plays a core role when examining consciousness in these patient populations. The combination of memory impairment and loss of awareness/wakefulness forms the basis of the operational definition of consciousness: Examination of an individual’s learning and memory function is impossible without the individual’s awareness/wakefulness. As such, awareness/wakefulness in combination with retained learning and memory are the two fundamental dimensions of consciousness, which are accessible to the observer. From a physiological point of view, there is a close relationship between these two phenomena. In TLE, even when the seizures remain focal, impairment, or even loss of consciousness may occur (Englot and Blumenfeld, 2009). Most importantly, the seizure onset zone in TLE is located in memory-relevant regions, such as the hippocampus and neighboring regions. During focal seizures with loss of consciousness, widely distributed slow rhythmic activity can be recorded over the temporal lobe (Gastaut, 1953), the frontal lobe (Williamson et al., 1985), and the neighboring regions. This ictal slow rhythmic activity resembles to a certain degree the slow waves during sleep also in terms of global efficiency, measuring facilitation of information transmission (Gast et al., 2014). It may be speculated that in both cases, deep sleep and peri-ictal loss of consciousness, slow-wave activity is related to impairment or loss of consciousness. In the network inhibition hypothesis, Englot and Blumenfeld (2009) proposed that slow activity in the neocortex occurring during a seizure is responsible for loss of consciousness in TLE patients. These slow waves are supposed to reflect the ictal activity in the limbic system and its functionally connected regions. Englot and Blumenfeld’s theory states that focal seizures spread to the thalamus, disrupting corticothalamic interactions and also suppressing arousal systems. Several mechanisms are discussed in these respects. For example, the thalamic reticular nucleus and the lateral septum are usually a mediator of excitatory activity for the neocortex (Steriade et al., 1997). During temporal lobe seizures, spikes in the hippocampus activate these subcortical activation systems abnormally, so that they inhibit subcortical structures and the ascending reticular activation system, leading to decreased excitation of the neocortex. The hypothesis is supported by intracranial EEG-findings of high synchronization between the thalamus and association cortices during seizures (Arthuis et al., 2009).

*Could the same mechanism, which mediates loss of consciousness, also contribute to the washout of stored memories? Are slow waves a correlate of this mechanism?* Slow waves occur in conditions of suppressed memory function in temporal lobe epilepsy. In the intracarotid sodium amobarbital test, the presence of slow waves indicated pharmacological action of the injected barbiturate, which is sufficient to result in memory impairment (Jones-Gotman et al., 1994). Similarly, Vuilleumier et al. (1996) report a case of a woman who experienced repeated status epilepticus with severe transient amnesia, accompanied by rhythmic epileptic spikes and waves at 3.5–4 Hz in the EEG. However, as soon as the condition ended, the women could recall everything and her cognitive performance was normal. Thus, slow-wave components of the spike–wave complex may have a deactivating function, such as blocking of memory retrieval or interference with memory consolidation. Until today, this potential effect of slow waves on memory function has never been examined in prospective studies. In addition, its relationship to the occurrence of loss of consciousness is far from understood.

## 3 Structural Brain Damage, Seizures, or Ictal Slow Waves?

*Is memory affected by structural brain damage, which eventually causes seizures, or is memory impairment a result of the seizures, being possibly related to the occurrence of ictal slow waves?* The authors support the view that it is highly likely that both possibilities are eligible, but the amount of contribution of one or the other of these two processes may vary with epilepsy subtype, its age of onset, localization, lateralization, time-scale of the affected memories, age, type, and number of seizures – especially the occurrence of generalized tonic–clonic seizures, status epilepticus, and discharges during slow-wave sleep (Elger et al., 2004; Stefan and Pauli, 2008).

To discuss this section’s question, we performed a systematic literature search on PubMed (accessed on 10th of April, 2015) with the search terms *epilepsy AND seizure AND memory AND (slow waves OR delta OR sleep)*, yielding 123 articles. Of these, 103 were excluded based on the title, 8 based on the abstract, and 2 based on the full text. Thus, 10 articles were included into this review. In addition, we searched the references in these articles in order to include further articles. We list those which refer to peri-ictal slow waves in Table 1 and discuss the others in the following sections.

**Table 1:** **Research on epilepsy, cognitive impairment, and peri-ictal slow waves**.

Reference	*N*	Epilepsy or seizure type	Slow-wave context	Structural brain damage	Results/cognitive outcome
Bower et al. (2015)	6	MTLE	Post-ictal	Normal/HC atrophy	Post-ictal slow waves strengthen (*consolidate*) seizure networks
Thompson and Duncan (2005)	136	Focal (*N* = 125) or generalized (*N* = 11) epilepsy	Seizure type	54% focal and/or global atrophy	Cognitive decline with frequent generalized tonic–clonic seizures over 10 years
Quiroga et al. (2002)	8	TLE	Tonic–clonic seizures	–	Dominant EEG-frequency of tonic–clonic seizures is <4 Hz
Dodrill (2002)	Review	–	Seizures	–	Cross-sectional studies, but not longitudinal studies found cognitive impairment being related to seizure frequency

Cross-sectional studies according to Dodrill (2002)					

Jokeit et al. (2000)	37	TLE	Seizures with loss of consciousness	–	Intellectual loss in patients with refractory partial seizures correlates with duration of epilepsy
Trimble (1988)	40	Seizure types: tonic–clonic, focal with loss of consciousness spike–wave	Tonic–clonic seizures	–	A loss of more than 15 IQ points is related to more generalized tonic–clonic seizures
Dodrill (1986)	94	All types, excluding status	Tonic–clonic seizures	–	A lifetime number of >100 generalized tonic–clonic seizures result in diminished cognitive capacity
Dikmen and Matthews (1977)	72	Tonic–clonic seizures	Tonic–clonic seizures	–	Higher frequency of tonic–clonic seizures, early onset, and long duration is related to cognitive impairment
Dodrill and Troupin (1976)	2	Tonic–clonic and other generalized seizures	Tonic–clonic	–	Twin study: the twin with more generalized tonic–clonic seizures showed significantly impaired intellectual abilities (memory)
Lennox and Lennox (1960)	1471	All types	Tonic–clonic seizures	–	Mental impairment is increased with seizure number and occurrence of generalized tonic–clonic seizures

*(M)TLE, (mesial) temporal lobe epilepsy; HC, hippocampus*.

Vingerhoets (2006) reviewed the current state of research with respect to the possible impact of seizures on cognitive functions. The author referred to four longitudinal studies in children, which were summarized by Dodrill (2004). All examined children (*N* = 11–83) with a test–retest period of 1.5–4.2 years, showing a decline of intelligence in symptomatic generalized epilepsies, especially those with myoclonic seizures. By contrast, the situation seems to be less clear in adults [studies with *N* = 9–102 were reviewed in Dodrill (2004) and Vingerhoets (2006)]. Vingerhoets criticizes that the search for seizure-related variables mediating cognitive decline is confounded by so many factors, that valid conclusions can rarely be drawn from studies. Specifically, he claims that in localization-related epilepsy cross-sectional studies are not able to distinguish the effects of the underlying lesion and the eventual long-term effects of epilepsy.

In patients with generalized tonic–clonic seizures, memory impairment of varying degrees is frequently reported. Thompson and Duncan (2005) performed a longitudinal examination of at least 10 years in severe intractable epilepsy. In this study, the frequency of generalized tonic–clonic seizures contributed most to the decline in verbal intelligence, performance intelligence, verbal recall, and naming. Quiroga et al. (2002) analyzed secondarily generalized tonic–clonic seizures with short time Fourier Transform from scalp EEG at electrode C4 and found that these seizures start with activity at about 8 Hz in the tonic phase, which afterwards slows down to 1–2 Hz. The dominance of slow waves in generalized tonic–clonic seizures and the correlation of seizure frequency and memory decline may raise speculation about the relevance of mechanisms that cause slow waves for the cognitive decline in these patients.

Table 1 shows that cross-sectional studies provide evidence for cognitive impairment in patients with generalized clonic tonic seizures. Some longitudinal studies in adult and mixed populations provide evidence for progressive intellectual decline in patients with (secondarily) generalized tonic–clonic seizures (Dodrill, 1986; Stefan and Pauli, 2002), but not for patients with focal seizures with or without loss of consciousness (Holmes et al., 1998; Stefan and Pauli, 2002; Griffith et al., 2007; Helmstädter and Elger, 2009). However, a problem in examining the impact of seizures is that most studies are based on self reporting. Self reporting may miss up to half of the seizures (Hoppe et al., 2007), especially when seizures involved the left temporal lobe (Kerling et al., 2006) and when loss of consciousness occurs (Detyniecki and Blumenfeld, 2014). In the rat model, occurrence of 69 or more secondary generalized tonic–clonic seizures resulted in neuronal loss in the temporal hilus of the dentate gyrus, CA1 and CA3 of the hippocampus (Kotloski et al., 2002). Due to these progressive, permanent functional and structural abnormalities, spatial memory deficits became evident. The abnormalities arise from apoptotic cell death, which is already detectable in brief and intermittent seizures (Sutula and Pitkänen, 2002).

Is underreporting the reason why cross sectional, and not longitudinal studies find a relation between secondary generalized tonic–clonic seizures and cognitive impairment? Helmstädter and Elger (2009) support the view that in TLE memory impairment occurs at an early age, but then, cognitive development runs in parallel with healthy populations. Therefore, cross-sectional studies find a difference, but longitudinal studies do not.

There is an additional way to explain this discrepancy. It is possible that memory impairment is a result of disruption of encoded contents. This disruption may not cause progression of memory impairment, but a constant impairment of memory due to the interference of uncontrolled seizures. This could explain why most cross-sectional studies reveal an impact of generalized tonic–clonic seizures, but only a few longitudinal studies (Dodrill, 2002). Jokeit et al. (2001) corroborate this assumption. They found that, in patients with left-sided TLE, the recall of learned content was impaired when a seizure occurred within 24 h of learning.

We consider the memory effects of generalized tonic–clonic seizures of high importance (Dodrill, 2002). In these seizures, slow waves and memory decline co-occur. In addition, according to the results of Jokeit et al. (2001), a short-term effect of seizures on memory entries exists. It is likely that this effect is reversible when seizures are under control. In 1968, Rodin (1968) found that patients with good seizure control showed increases on the Wechsler–Bellevue intelligence scale. A similar finding was reported by Seidenberg et al. (1981). Future research needs to address the question whether this short-term effect is measurable regardless of the type of the seizure or if it is limited to certain subtypes – for example, seizures with peri-ictal slow waves.

## 4 Slow Waves During Sleep, Seizures, and Memory Problems

In the spiritual cantata *“Ich will den Kreuzstab gerne tragen” (“I will the cross-staff gladly carry”)* of Johann Sebastian Bach (BWV 56), death is called the brother of sleep *(“Komm, o Tod, du Schlafes Bruder”, Come, O death, of sleep the brother”)*. While the behavioral resemblance of sleep and death is somewhat understandable, we would rather say that the brother of sleep is the seizure, in agreement with Gast et al. (2014) who states that a seizure is much like condensed sleep. Indeed, there is a striking similarity between ictal slow waves, which occur in relation to loss of consciousness and the EEG that can be recorded during deep sleep, that is, slow-wave sleep (Englot and Blumenfeld, 2009).

Gast et al. (2014) assessed the functional networks during seizures and sleep by calculating global efficiency of the scalp EEG and found that the networks progressively disconnect over the course of both seizures and physiological sleep. In other words, the deeper the sleep stage is, the less connected are the functional networks. Similarly, the brain encounters disconnection over the course of a seizure, which is most severe at the stage of post-ictal slow-wave activity. It has been suggested that the same neuronal circuits may produce epileptiform activity and slow oscillations during sleep (Steriade et al., 1993a,b; Beenhakker and Huguenard, 2009).

Since post-ictal slow waves are much like deep sleep, we should not automatically assume that peri-ictal slow waves always correlate with disrupted memory. Instead, on the background that memory consolidation occurs during slow-wave sleep stages (Gais and Born, 2004a), and that the underlying processes of slow waves coordinate this consolidation (Mölle and Born, 2011), we could also speculate that the opposite may be true and epilepsy-related slow waves could indeed occur during memory consolidation. Consolidation in slow-wave sleep is the replay of neuronal firing patterns similar to those observed during learning (McGaugh, 2000). In that sense, Bower et al. (2015) claimed that post-ictal slow-wave sleep, but not wakefulness, caused consolidation. Unlike the physiological learning mechanism, this seizure-related consolidation consisted in reactivation of seizure-related neuronal activity. In other words, post-ictal slow waves replay – or *consolidate* – seizure-generating mechanisms.

Thus, the functional difference between functional consolidation during slow-wave sleep and post-ictal slow waves lies in differential activation of networks. While the replay of memory entries in the neocortex during slow-wave sleep are supposed to follow a certain pattern (Gais and Born, 2004a), the activations, which co-occur during peri-ictal slow waves, could be unspecific and chaotic – much like seizures themselves. Analogously, spike–waves have been interpreted as the änti-binding” or ünlearn” process, which washes out eventual false associations, caused by hyper-synchronous hippocampal–neocortical gamma oscillations at the onset of epileptiform discharges (Medvedev, 2001).

In children with BECTS, who have abundant interictal epileptiform discharges during non-REM sleep, declarative short- and long-term memory deficits may be caused by impaired sleep-related memory consolidation (Verrotti et al., 2014). While the resulting deficits are persistent, children with interictal epileptiform discharge occurring during wakefulness, experience transitory neuropsychological deficits. The mechanism behind sleep-related impairment is possibly such that the spikes and waves during slow-wave sleep interfere with normal downscaling of slow-wave activity during sleep, which is crucial for mediating long-term storage of memories (Gais and Born, 2004a). Thus, it is plausible to expect memory decline when patients fail to reach the stage of slow-wave sleep.

Urbain et al. (2011) reported that in contrast to healthy children who benefit from sleep for memory consolidation (Ashworth et al., 2014), recall performance of studied word pairs decreased overnight in children with BECTS. Improving the sleep EEG by hydrocortisone treatment also normalized overnight memory performance. A similar lack of consolidation during sleep was reported in children with idiopathic focal epilepsies with frequent interictal epileptiform discharges (Gaier et al., 2015). Similarly, Sud et al. (2014) reported that children with epilepsy did not benefit more from sleep than from wakefulness for memory consolidation. This relationship between nocturnal spike activity and memory consolidation could also be the cause for the neuropsychological deficits in encephalopathy with status epilepticus during slow-wave sleep. Tassinari et al. (2009) hypothesize that altered cortical plasticity is the main reason for impaired learning in these syndromes. As such, the authors regard the severe cognitive deficits as direct consequence of continuous epileptic activity during slow-wave sleep. However, especially in epilepsy syndromes with frequent hippocampal interictal spikes the negative impact of these sleep-independent events on cognition (Kleen et al., 2010, 2013; Fitzgerald et al., 2013) needs to be disentangled from disturbed slow-wave sleep (Bazil, 2002; Parisi et al., 2010; Chan et al., 2011).

The results from intracranial EEG by Axmacher et al. (2008a,b) support the current view that during wakefulness new information passes the entorhinal cortex and proceeds to the hippocampal CA3 region for temporary storage. During sleep, the information flow is reversed and the information flows back from the hippocampus to the neocortex (Buzsáki, 1989, 1996; McClelland et al., 1995; Qin et al., 1997; Hasselmo, 1999; Mölle et al., 2002, 2006; Sirota et al., 2003). Slow oscillations might coordinate the hippocampo-neocortical dialog by driving ripple activity. Thus, in intracranial recordings of the hippocampus, more ripples can be detected during the positive slow-oscillation half wave (Mölle and Born, 2011). The positive half-wave of slow oscillations in the EEG is the correlate of a depth-negative extracellular field potential, and thus, of cortical depolarization (Gais and Born, 2004a). This depolarized, excitatory state stimulates both spindles and high-frequency oscillations, while the hyperpolarized, inhibitory state suppresses them (Mölle et al., 2002).

Reviewing the role of slow-wave sleep for memory consolidation is well beyond the scope of the present review. By referring to the vast literature on research about sleep and memory, we agree that the importance of slow-wave sleep for memory consolidation is well established. In their review, Gais and Born (2004a) suggest that slow waves drive simultaneous activation of cortical neurons from thalamic spindles and hippocampal ripples leading to consolidation of memory entries. Therefore, fundamental differences distinguish slow-wave sleep and peri-ictal slow waves. These differences let peri-ictal slow waves be pathologic, while slow waves during sleep are a physiologic activity. Bower et al. (2015) suggest that post-ictal slow waves *hijack* physiological consolidation mechanisms to strengthen the epileptic networks.

In sum, slow-wave sleep and post-ictal slow activity can be distinguished from an electro-morphological point of view, the localization, as well as the synchronization patterns. Slow-wave activity in the post-ictal state of is a global spectral shift to slower frequencies, but regional spatial shifts in slow-wave activity is possible and depends on the type of seizure (Yang et al., 2012). For example, in tonic–clonic seizures, the EEG-activity slows down over the course of the seizure, ending up in the bilaterally symmetrical and synchronous post-ictal slow waves, which then usually increase in frequency until reaching the normal state (Chokroverty and Thomas, 2013). By contrast, in focal seizures with loss of consciousness and secondarily generalized seizures, delta power decreases in the ipsilateral temporal region but increases in other regions, especially the frontal region (Yang et al., 2012). In this context, it was postulated that there exists a post-ictal network, which includes temporal, lateral–frontal, prefrontal and dorsal–medial–frontal regions, and spreading also to parietal regions. By contrast, slow waves during sleep are typically polymorphic or semirhythmic; it may occur mostly on anterior regions with sinusoidal morphology around 1.5–2 Hz or posterior and polymorph around 1 Hz (Wehrli and Loosli-Hermes, 2003).

Moreover, neurobiological research revealed that the role of cholinergic circuits might be of relevance for the distinction of different mechanisms of slow-waves. In the model of Hasselmo (1999), acetylcholine inhibits feedback between hippocampal regions and between hippocampal and neocortical areas during wakefulness, so that encoding of new memory entries takes place. By contrast, during slow-wave sleep, low-cholinergic levels facilitate the replay of memories. High-cholinergic levels during sleep, as obtained by injection of the cholinesterase inhibitor physostigmine, interfere with sleep-dependent consolidation (Gais and Born, 2004b). A similar relationship exists with cortisol (Steriade, 1994), being high during wakefulness and low during sleep, and enhancing memory formation but preventing recall of old memory entries during wakefulness. Cholinergic and adrenergic systems both block the typical delta rhythm during slow-wave sleep and the slow oscillation below 1 Hz, which is known to drive sleep spindles. By contrast, certain subtypes of epilepsy, specifically absence epilepsy and nocturnal frontal lobe epilepsy, may be associated with acetylcholine receptor mutations, which affect the arousal system and lead to micro-arousals during non-REM sleep (Ferini-Strambi et al., 2012; Halász et al., 2013). Moreover, it was suggested that acetylcholinergic neurons are lesioned in refractory epilepsy (Hayashi et al., 2012).

## 5 The Interaction of Slow Waves and Ripples During Memory Consolidation

Seizure- or spike-related high-frequency oscillations, so-called ripples (80–200 Hz), in the hippocampus and ripples that are coupled to phases of slow oscillations during memory consolidation (Kerber et al., 2014) might also mirror the complex relationship between physiological slow waves during deep sleep and pathological peri-ictal slow waves (Gast et al., 2014).

Intracranially recorded ripples are linked to memory consolidation in the hippocampus and the rhinal cortex during wakefulness (Axmacher et al., 2008a) and sleep (Clemens et al., 2007). Paradoxically, ripples are also regarded as a pathologic finding in epilepsy and are indicative of the seizure onset zone (Worrell and Gotman, 2011; Engel and da Silva, 2012; Jacobs et al., 2012; Jette and Wiebe, 2013; Staba et al., 2014). It is difficult to determine whether ripples in epileptic patients reflect epileptic or regular physiological activity (Clemens et al., 2007). It has been suggested that ripple patterns could be distinguished based on frequency. Faster oscillations are thought to be pathologic (Bragin et al., 1999). Furthermore, background oscillations may be used to distinguish pathological from physiological activity (Kerber et al., 2014). Dümpelmann et al. (2015) found that pathologic ripples occur mainly in the seizure onset zone, but independent from the sleep-wake cycle, whereas physiologic ripples in the temporal area can be detected mainly during sleep. Frauscher et al. (2015) found that spikes and high-frequency oscillations during sleep occurred mainly at the transition from high-amplitude slow waves to low-amplitude states. By contrast, physiological activity occurred mainly during high-amplitude slow waves. Clemens et al. (2007) described an association between intrahippocampal ripples and slow waves during sleep and additionally found that there is a time locked close association between ripples and interictal epileptic spikes. The authors state that the spike–ripple phenomena could be seen as an epileptic version of the normal sharp wave–ripple complex. Most interestingly, in their study, the driving role of the neocortical slow oscillations was hampered inpatients with structural abnormalities of the mesial temporal lobe. Therefore, the authors concluded that these alterations might contribute to deficits in sleep-related memory consolidation and long-term memory as shown in earlier studies (Blake et al., 2000; Clemens et al., 2003).

**Figure 1:**
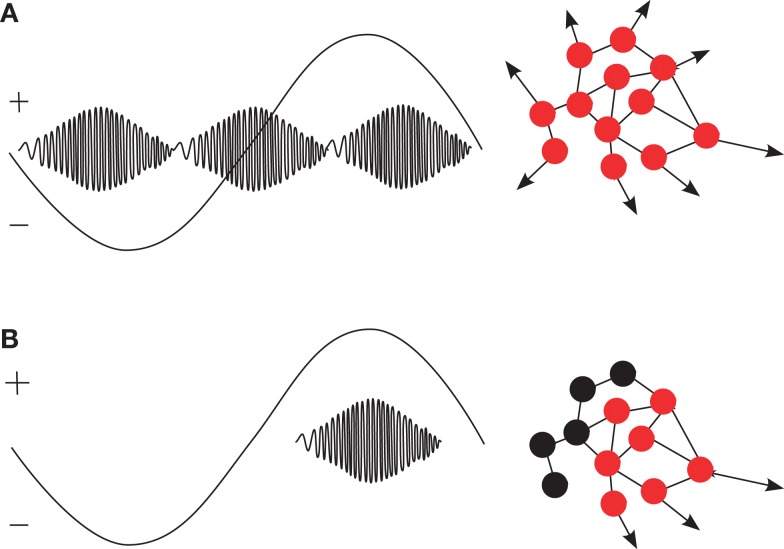
**(A)** In patients with structural abnormalities in the hippocampus, ripples occur in both the positive and negative peak of hippocampal delta oscillations (Clemens et al., 2007). In analogy to the generation of spikes (Karlócai et al., 2014), we hypothesize that pathological ripples are caused by a simultaneous increase of the firing rate in large populations of (pyramidal) cells (red dots) because of inhibition breakdown. **(B)** Under normal conditions, functional ripples occur mostly in the excitatory, positive half of slow oscillations and are relevant for memory consolidation (Gais and Born, 2004a; Clemens et al., 2007; Axmacher et al., 2008a). We hypothesize that activity from inhibitory neurons (represented as black dots) is necessary to generate selective firing patterns.

Karlócai et al. (2014) suggested that epilepsy-related activity results from all neurons increasing their firing rate compared to physiological ripples. Thus, the similarity between physiological ripples and interictal spikes accompanied by ripple-like oscillations can be interpreted as different points of the same continuum. Extending the ideas arising from the results of Clemens et al. (2007), Karlócai et al. (2014), and Frauscher et al. (2015), we hypothesize that the driving effect of slow oscillations interacts with pathological changes. Under normal conditions, the depolarized state, i.e., the positive half-wave of the slow oscillation facilitates the occurrence of physiologic ripples while the hyperpolarized state inhibits them (Gais and Born, 2004a). Pathological changes jeopardize the interplay of excitation and inhibition so that uncontrolled excitation can occur in any phase of the slow oscillation. As a result, ripples occur in both the excitatory and inhibitory half-wave of the hippocampal delta oscillations or even without any background activation. In other words, we assume that slow oscillations in epilepsy occur under damaged network-properties, leading to breakdown of inhibition and, thus, unsystematic generation of pathological ripples. Figure 1 illustrates this idea.

However, there exists interindividual variance in the driving effect of slow oscillations for memory-consolidating ripples. In a neurofeedback study, Fritz et al. (2011) found that feedback-induced slow cortical potentials are related to hippocampal spiking activity, but whether spikes increased or decreased with slow positive or negative shifts varied between subjects. This variance might be due to the between-subject variation of the response to neurofeedback training, or to a general between-subject variation of the phase-amplitude coupling. Therefore, it is necessary that future studies confirm the driving role of slow oscillations on a single-subject level.

## 6 Conclusion

In this narrative, and one may say also speculative review we examined the literature on possible relationships between slow-wave activity in the EEG and memory performance in patients with epilepsy. Specifically, we posed the question of whether it is possible that mechanisms, which produce ictal slow waves also negatively, influence memory performance in epilepsy. In sum, four possible relations between the occurrence of slow waves in the EEG of patients with epilepsy and memory should be addressed in the future by prospective studies:

1. First, peri-ictal slow-wave activity coincides with loss of quantitative consciousness. While ictal slowing of brain activity (i.e., slow frequencies increase in the EEG) in focal seizures is detectable during loss of consciousness, there is less evidence, that this phenomenon correlates with memory deficits. By contrast, frequent generalized tonic–clonic seizures are indeed related to cognitive impairment in severe intractable epilepsy. The short-tonic phase starts with alpha oscillations, but the prolonged clonic phase is driven by slow activity. *Future research should assess whether tonic–clonic seizures delete previously learned content*.
2. Second, much like the mechanism, which causes slow-wave activity supposedly also mediates breakdown of consciousness, the findings of simultaneous amnesia and slow-wave activity suggest that the processes underlying slow oscillations may indeed interfere with access to memory functions at a short range of time. In addition, an interference with consolidation of memories is likely to occur in several epilepsy syndromes.*Future projects should examine whether seizures with or without occurrence of slow waves have a short-term impact on memory*.
3. The third relationship can be found in slow waves during deep sleep. In some types of epilepsy, sleep-related memory consolidation seems to be disturbed. The resulting memory problems are most likely reversible if normal sleep can be restored, which is in contrast to memory deficits because of structural changes in chronic epilepsy.*It needs to be assessed in which types of epilepsy insufficient sleep-related memory consolidation is present and if control of seizures eliminates the related cognitive deficits*.
4. Fourth, under normal conditions, memory consolidation during slow-wave sleep co-occurs with ripples, which are phase-locked to the positive peak of the slow oscillation. It is of interest of whether a disturbance of this cross-frequency coupling during sleep, and wakefulness, or in the peri-ictal period is related to erroneous consolidation of memories in specific types of epilepsy. *Future research should disentangle the possible interference of pathologic ripples during encoding, consolidation, and retrieval of memory content from the physiologic coupling of ripples and slow oscillations during consolidation processes occurring in slow-wave sleep*.

## Author Contributions

Conception and decision on which references to include revising and approving of this work were carried out by all authors. The first author wrote the manuscript in accordance with the intellectual input of both authors. Both authors agree to be accountable for all aspects of the work in ensuring that questions related to the accuracy or integrity of any part of the work are appropriately investigated and resolved.

## Conflict of Interest Statement

The authors declare that the research was conducted in the absence of any commercial or financial relationships that could be construed as a potential conflict of interest.
